# Mechanism of liposome arsenic trioxide regulating tumor microenvironment and sensitizing tumor immune response after radiofrequency ablation

**DOI:** 10.3389/fimmu.2026.1776060

**Published:** 2026-06-29

**Authors:** Chao Liang, Shuguang Ju, Hao Li, Daqian Han, Jiacheng Wang, Yangyang Niu, Jianzhuang Ren, Xinwei Han, Xuhua Duan

**Affiliations:** Department of Interventional Radiology, The First Affiliated Hospital of Zhengzhou University, Zhengzhou University, Zhengzhou, China

**Keywords:** arsenic trioxide, carcinoma, hepatocellular, immunotherapy, liposome, radiofrequency ablation

## Abstract

**Objective:**

We hypothesized that preoperative administration of liposome arsenic trioxide (LATO) would downregulate hypoxia-inducible factor-1α (HIF-1α) in residual tumors following incomplete radiofrequency ablation (iRFA), thereby modulating the tumor microenvironment, suppressing residual tumor proliferation and angiogenesis, and consequently enhancing the antitumor efficacy of programmed death-ligand 1 (PD-L1) blockade.

**Methods:**

Using a murine Hepa1–6 hepatocellular carcinoma model subjected to incomplete radiofrequency iRFA, cohorts were stratified based on preoperative LATO administration and postoperative anti-PD-L1 antibody combination therapy, and the indexes such as proliferation and immune infiltration of residual tumor were tested respectively.

**Results:**

After iRFA, residual tumors exhibited significant up-regulation of PCNA, CD31, and HIF-1α compared with the control group (all *p* < 0.0001). Intravenous infusion of LATO before RFA could significantly decrease the expression of PCNA, CD31 and HIF-1α in the residual tumor, promote the infiltration of immune cells (CD4^+^, CD8^+^) and prolong the survival time of mice. After RFA, combined use of anti-PD-L1 antibody can further reduce the expression of cell proliferation and tumor proliferation.

**Conclusion:**

Pre-treatment with LATO prior to RFA appears to modulate the tumor microenvironment, which may contribute to improved ablation efficacy and help restrain the progression of residual tumors. Furthermore, it could potentially enhance the antitumor effect of combining RFA with anti-PD-L1 antibody therapy by sensitizing the tumor immune response.

## 1 Introduction

Primary hepatocellular carcinoma (HCC) is the sixth most common cancer and the third leading cancer death worldwide in 2020 (1). Due to tumor size, number and poor liver function reserve, only 10 – 20% of HCC patients are suitable for surgical resection (2). Radiofrequency ablation (RFA) is a common clinical ablation method, which can radically treat HCC ≤ 3 cm in diameter and achieve the efficacy comparable to surgical resection (3). With the increase of RFA output energy and the safe application of multipolar needle, RFA can safely and effectively treat 3.0 – 5.0 cm nodular HCC and 5.0 – 7.0 cm large HCC (4). Incomplete tumor necrosis after RFA occurs when the tumor volume increases due to the different tissues within the tumor limiting energy deposition, increased heat loss due to increased blood flow within large tumor and the ablation volume decreasing exponentially with distance from the electrode due to current density (4). Incomplete necrosis is mainly concentrated around the blood vessels in the tumor and at the edge of coagulative necrosis, and the residual tumor cells at this site are the root cause of tumor recurrence and metastasis (5).

Kong et al (6) revealed that sublethal thermal stress from incomplete ablation upregulates hypoxia-inducible factor-1α (HIF-1α) in residual tumor tissues, promote angiogenesis in residual tumor tissues, and accelerate the progression of residual tumor tissues. Arsenic trioxide (ATO) was demonstrated to inhibit HIF-1α expression in residual HCC cells after iRFA, thereby inhibiting tumor progression (7). Shi et al (8) found that after RFA, residual tumors were able to up-regulate the expression of programmed cell death-ligand 1 (PD-L1), inhibit the function of CD4^+^ T and CD8^+^ T cells, and then rapidly suppress the immune response produced by RFA. Noman et al (9) found a positive correlation between PD-L1 and HIF-1α expression in tumor tissues, and blocking the HIF-1α pathway could inhibit PD-L1 expression; PD-L1 is a direct target of HIF-1α, which can block T-cell-mediated anti-tumor immune responses under hypoxia. RFA combined with immune checkpoint inhibitors (ICIs) in the treatment of liver metastases can synergistically enhance the tumor immune response induced by RFA, indicating a broad therapeutic prospect of this combination regimen (10). However, the presence of an immunosuppressive microenvironment leads to unsatisfactory objective response rates to ICIs in patients with hepatoma (11). How to improve the response rate to ICIs and break the tolerance of immunotherapy has become a difficult problem in HCC immunotherapy.

Intravenous administration of ATO can induce apoptosis and inhibit the growth of HCC cells (8, 12, 13). However, systemic administration of ATO for HCC treatment is limited by unfavorable pharmacokinetics and dose-limiting toxicities. Following intravenous infusion, ATO rapidly binds to serum proteins—predominantly hemoglobin—with high affinity, resulting in low plasma free drug concentrations and limited tumor tissue penetration (14). Clinically, intravenous ATO at the approved dose (0.15 mg/kg or 10 mg) achieves a mean peak plasma concentration (Cmax) of approximately 124 ng/mL for total arsenic, with substantial interpatient variability (coefficient of variation ~60%), and exhibits an effective elimination half-life of ~3 days, leading to progressive systemic accumulation with daily dosing (15). These pharmacokinetic characteristics restrict the achievable intratumoral drug exposure and necessitate prolonged hospitalization for intravenous infusion. Moreover, systemic ATO accumulation is associated with significant toxicities, most notably dose-dependent QT interval prolongation via hERG potassium channel blockade and increased cardiac calcium currents, which can precipitate torsades de pointes and sudden cardiac death (16). The underlying mechanisms involve excessive ROS generation, mitochondrial dysfunction, intracellular Ca^2+^ overload, and depletion of antioxidant defenses, rendering cardiomyocytes particularly vulnerable (16). To circumvent these limitations, liposomal drug delivery systems have been developed to alter the biodistribution of ATO. da Rosa (17) et al. prepared liposome-encapsulated arsenic trioxide (LATO) for acute promyelocytic leukemia treatment, demonstrating that liposomal encapsulation reduces systemic toxicity while maintaining therapeutic efficacy compared with conventional ATO solution. The liposomal formulation enhances drug deposition at tumor sites through the enhanced permeability and retention effect and protects the encapsulated drug from rapid protein binding and renal clearance, thereby improving the therapeutic index (17, 18).

This study intends to use LATO to treat mouse iRFA liver tumor model to explore whether the application of LATO can reduce the expression of PD-L1 in residual tumor cells by reducing HIF-1α, thereby enhancing the anti-tumor immune response after ablation, and explore whether LATO can change the tumor immune microenvironment after ablation through HIF-1α/PD-L1 axis, improve the immune response effect of ICIs, and finally synergistically increase the efficacy of RFA.

## 2 Materials and methods

### 2.1 Preparation of LATO

The liposome formulation was prepared using the thin-film hydration method. Briefly, pre-weighed lecithin and cholesterol (total lipids: 100 mg) were dissolved in 3 mL of chloroform containing vitamin E (1 mg/mL). A prescribed amount of ATO solution (in 0.1 mol/L NaOH) was then added, and the mixture was transferred to a 250 mL pear-shaped flask. The organic solvent was removed by rotary evaporation under reduced pressure in a 40 °C water bath to form a thin, uniform lipid film. This film was subsequently hydrated with a 40 °C hydration medium containing cryoprotectants, creating a crude suspension. The suspension was vortexed, gently shaken for 2 hours, and then subjected to ice-bath sonication, yielding a stable, milky-white L-ATO solution. Finally, small unilamellar vesicles with a particle size of 0.25-1 μm were obtained by applying specific sonication time and intensity, and the final preparation was stored at 4 °C. The characterization information of LATO is detailed in the **Supplementary Materials**.

### 2.2 Cell culture

Hepa1–6 cells, acquired from Procell Life Science & Technology Co., Ltd. (Wuhan, China), were cultured under standard conditions. Specifically, they were grown in high-glucose DMEM (Gibco, Thermo Fisher Scientific) enriched with 10% fetal bovine serum (Gibco), 100 U/mL penicillin and 100 µg/mL streptomycin, at 37 °C in a 5% CO_2_ incubator (19).

### 2.3 Cell invasion assay

After Hepa1–6 cells were in log-phase growth, hypoxia inducer (COCL_2_, 150 μmol/L) was added for 24h to induce hypoxia. Matrigel was added to the upper layer of transwell chamber beforehand. After matrigel solidified, the cells were diluted with serum-free medium of 0, 5, 10, 15 and 20 ng/ml LATO and ATO solutions, 2.5 × 10^4^ cells/well cells were added to the upper layer of transwell chamber, and 10% fetal bovine serum medium was added to the lower layer of transwell chamber. The cells were cultured for 24 h. Transwell kit was stained to detect cell invasion.

### 2.4 Cell scratch assay

When Hepa1–6 cells were in log-phase growth, hypoxia inducer(COCL_2_, 150 μmol/L) was added for 24h to induce hypoxia. The cells in hypoxia state were placed into a 6-well plate. When the cells adhered 85%, scratch sites were created with 200 µL tip. The scratch site was photographed at 0 h and 48 h, respectively. The percentage of cell migration (%) = (scratch area at 0 h - area at the corresponding time point)/scratch area at 0 h × 100%.

### 2.5 CCK8

When Hepa1–6 cells were in log-phase growth, a hypoxia inducer(COCL_2_, 150 μmol/L) was added for 24h to induce hypoxic conditions. The cells were then maintained under hypoxia, seeded into 96-well plates at a density of 1 × 10^4^ cells/well, and treated with culture medium containing 0, 5, 10, 15, and 20 ng/ml LATO or ATO for 0 and 24 hours. OD450 was detected by CCK8 to calculate the cell proliferation rate.

### 2.6 Establishment of a mouse hepa1–6 hepatocellular carcinoma subcutaneous tumor model

Male C57 BL/6 mice (6–8 weeks old and weighing 20–25 g) from the Laboratory Animal Center of Zhengzhou University School of Medicine (Zhengzhou, China) were maintained under pathogen-free conditions. The animal studies were approved by the Ethics Committee of Zhengzhou University (ZZU-LAC20220427). The animal experiments were conducted from July 2021 to June 2023.

Following at least one week of acclimation, each mouse was subcutaneously inoculated in the right dorsal flank with 1 × 10^6^ Hepa1–6 cells on day 0. Tumors were measured for long (a) and short (b) diameters using vernier calipers. Tumor volume was calculated using the formula: V = a × b^2^/2. Upon reaching a tumor volume of 150–200 mm³ on day 15, the mice were randomly allocated into their respective treatment groups using a computer-generated random number sequence. The humane endpoint for tumor-bearing mice was defined as tumor volume exceeding 2000 mm³ or signs of distress such as lethargy, weight loss >20%, or ulceration. All efforts were made to minimize suffering.

### 2.7 Tolerability of tumor-bearing mice to toxic side effects of LATO

A total of 25 tumor-bearing mice were randomly allocated into five groups (n = 5 per group): control, ATO (2 mg/kg), ATO (3 mg/kg), LATO (2 mg/kg), and LATO (3 mg/kg). The control group received no treatment, while the other groups were administered the corresponding ATO or LATO solutions via tail vein injection. In accordance with AVMA guidelines, animals were euthanized by cervical dislocation at 1-, 3-, 7-, and 14-days post-injection. Intact specimens of liver, bile, kidney, left lower limb muscle, heart, and lung were collected via laparotomy for gross and pharmacokinetic evaluation. Serum levels of aspartate aminotransferase (AST), alanine aminotransferase (ALT), serum creatinine (Scr), and blood urea nitrogen (BUN) were measured using standard enzymatic assays before injection and on days 1, 3, and 7 to assess hepatic and renal function.

### 2.8 *In vivo* animal studies

Following anesthesia with 2.5% isoflurane under oxygen (0.8–1.0 L/min), mice were placed prone and secured on electrode pads to ensure direct contact. A sterile syringe was used to puncture the skin approximately 1 cm from the tumor edge. Sterile saline-soaked gauze was placed around the tumor to prevent skin burns during ablation. The RF generator was set to an upper power limit of 5 W and an upper temperature limit of 70 °C. A monopolar RF probe (17 G) was then inserted into the tumor center along its longitudinal axis. When the needle-tip temperature reached 45 °C, timing commenced for a 4-min ablation. Post-RFA, the needle track was ablated to minimize bleeding. Mice were recovered in warmed cages until fully conscious.

To evaluate whether LATO enhances RFA efficacy and modulates the tumor microenvironment, 40 tumor-bearing mice were randomized into four groups (n = 10 per group): (1) NC (untreated control); (2) iRFA alone; (3) iRFA + ATO (ATO injected via tail vein 12 h before iRFA); (4) iRFA + LATO (LATO injected via tail vein 12 h before iRFA). Tumor size was measured every two days. From each group, five mice were monitored for survival; the remaining five were euthanized by cervical dislocation on day 11 post-RFA for tumor collection.

To assess whether LATO infusion combined with anti-PD-L1 synergistically enhances the anti-tumor immune response post-iRFA, 50 tumor-bearing mice were randomized into five groups (n = 10 per group): (1) NC; (2) iRFA alone; (3) iRFA + anti-PD-L1 (200 μg mouse anti-PD-L1 antibody i.p. one day after RFA, then every other day for four doses); (4) iRFA + LATO (LATO i.v. 12 h before iRFA); (5) iRFA + LATO + anti-PD-L1 (LATO i.v. 12 h before iRFA, plus anti-PD-L1 as in group 3). Tumor size was recorded every two days. Five mice per group were followed for survival; the other five were euthanized on day 11 for tissue harvest. To minimize observer bias, the investigators who performed tumor volume measurements (caliper) and survival monitoring were blinded to group allocation.

### 2.9 RT-qPCR

Total RNA was extracted from tumor tissues with TRIzol reagent (Beijing ComWin Biotech) following the manufacturer’s protocol. The purity and concentration of the isolated RNA were then quantified using a NanoDrop spectrophotometer (Thermo Fisher Scientific). 2 μg RNA (Vazyme Biotech Co., Ltd) was reverse transcribed into cDNA using the First Strand cDNA Synthesis Kit according to the manufacturer ‘s instructions. After dilution of cDNA in Tris-EDTA buffer, real-time polymerase chain reaction was performed on a QuantStudioTM 3 (Thermo Fisher Scientific) system. Quantitative PCR (qPCR) reactions were performed in duplicate using the Taq Pro Universal SYBR qPCR Master Mix (Vazyme Biotech Co., Ltd) with specific primers. The amplification was carried out on all samples, using Rps18 as the endogenous control for normalization. Relative gene expression levels were calculated by the 2^−ΔΔCt^ method. The sequences of all primers used are listed in **Table 1**.

**Table 1:** Primers for real-time PCR.

Genes or binding sites	Primers (5’-3’)
Rps18(m)-forward	TGGGAAGTACAGCCAGGTTC
Rps18(m)-reverse	AGTGGTCTTGGTGTGCTGAC
Cth(m)-forward	GGGTCTTGCTGCCACCATTA
Cth(m)-reverse	GTACCTGTTGGTGCCTCCATA
Gclm(m)-forward	ACAATGACCCGAAAGAACTGCT
Gclm(m)-reverse	ACCCAATCCTGGGCTTCAAT
Hmox1(m)-forward	ACCTTCCCGAACATCGACAG
Hmox1(m)-reverse	CAGCTCCTCAAACAGCTCAATG

### 2.10 Immunohistochemistry

Following fixation in 4% paraformaldehyde, tumor tissues were paraffin-embedded and sectioned at a thickness of 4 μm. Consecutive sections were subjected to conventional hematoxylin and eosin (H&E) staining or immunohistochemical (IHC) staining. For IHC, sections were incubated with primary antibodies against CD4 (1:200, GB115428-100; Servicebio), CD8 (1:200, GB114196-100; Servicebio), PCNA (1:200, GB11010-100; Servicebio), CD31 (1:200, GB11063-2-100; Servicebio), and HIF-1α (1:200, GB111339-100; Servicebio), followed by DAB development and hematoxylin counterstaining. All IHC images were acquired under standardized conditions using an Olympus BX53 microscope equipped with a DP74 camera at 400× magnification with fixed exposure and illumination settings. For each sample, five fields were selected by systematic random sampling (predetermined grid pattern, avoiding necrotic regions and tissue edges) to minimize selection bias. The investigator was blinded to group allocation during both image capture and quantification. The quantification metric for each marker was chosen based on its biological nature: positive cell percentage/count was used for PCNA, CD4, and CD8 to enumerate discrete proliferating or infiltrating cell populations; cumulative optical density (IOD) was used for HIF-1α and CD31 to capture graded protein expression intensity and continuous vascular network density, respectively. Digital images were analyzed using ImageJ (version 1.53; National Institutes of Health). Following color deconvolution (H DAB vector), the DAB channel was extracted for each marker. For PCNA, CD4, and CD8, positive cells were enumerated using the ‘Analyze Particles’ function after automated thresholding (Otsu’s method) with size (10–100 μm²) and circularity (0.5–1.0) constraints. For HIF-1α and CD31, cumulative optical density (IOD) was measured after background subtraction (rolling ball radius = 50 pixels) and automated thresholding. Five non-overlapping fields per tumor section were analyzed, and the mean value per animal was used for subsequent statistical analysis. The number of positive cells or the cumulative optical density was quantified using ImageJ software, and the mean values from these replicates were used for subsequent statistical analysis.

### 2.11 Data analysis

**Figure 1:**
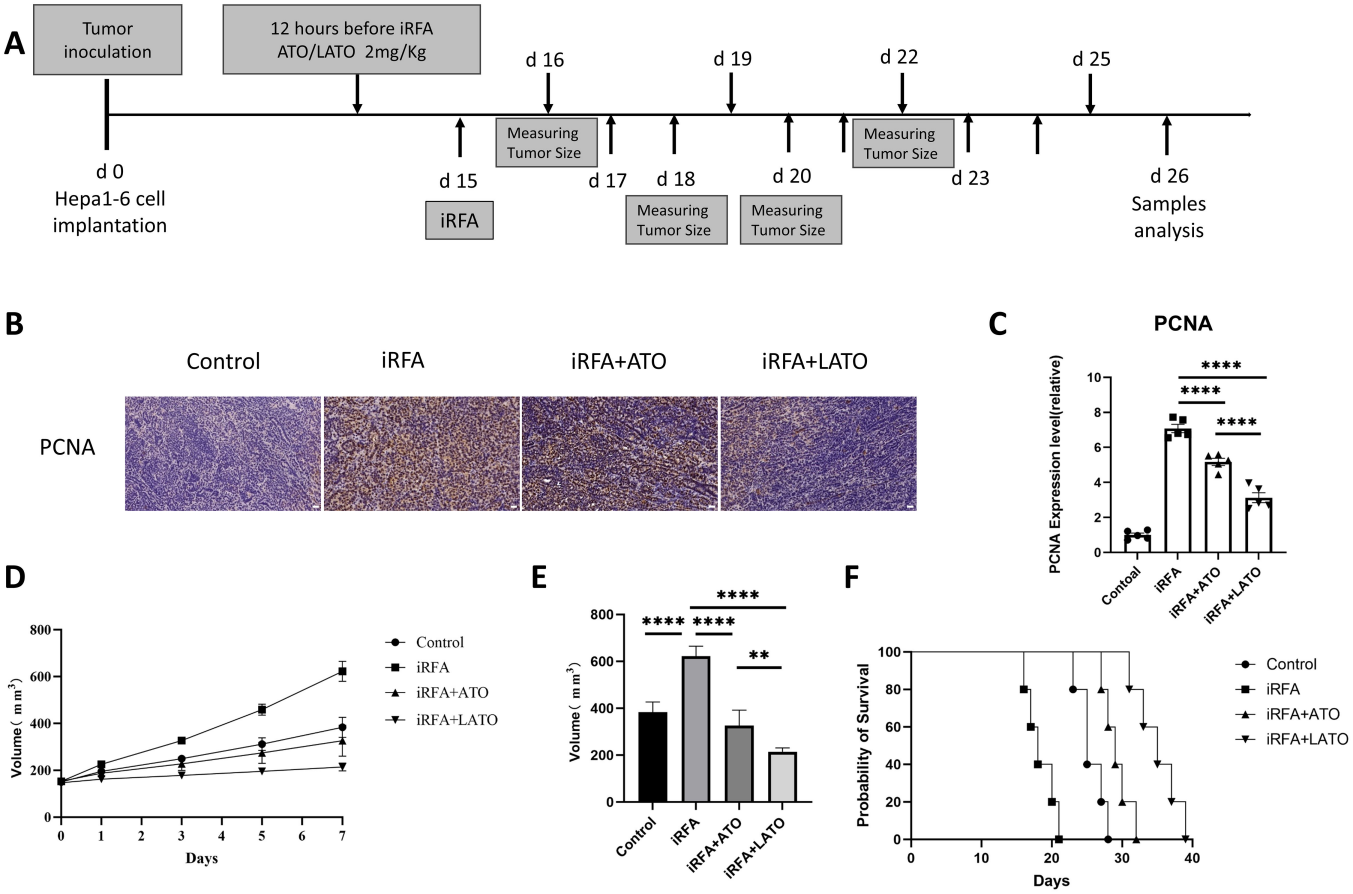
Administration of LATO suppresses tumor proliferation following iRFA and enhances survival in tumor-bearing mice. **(A)** Schematic diagram illustrating the experimental design for evaluating the effects of LATO on residual tumors post-iRFA. **(B)** Representative immunohistochemical (IHC) images of PCNA staining across the four experimental groups (scale bar = 20 μm). **(C)** Quantitative analysis of the percentage of PCNA-positive tumor cells in the four experimental groups. Statistical significance was determined by one-way ANOVA followed by Bonferroni’s multiple comparisons test. **(D)** Tumor growth curves in four groups post-iRFA. Tumor volumes were analyzed by mixed-design repeated-measures ANOVA with Group (4 levels) as the between-subject factor and Time (days 0, 1, 3, 5, 7) as the within-subject factor. A significant main effect of Group was found [F(3,16)=68.059, *p* < 0.001, partial eta squared=0.927], together with a significant Group x Time interaction [F(12,64)=61.700, *p* < 0.001, partial eta squared=0.920]. At day 7 post-treatment, tumor volume in the iRFA+LATO group (214.1 ± 15.3 mm3) was significantly lower than that in the iRFA group (622.0 ± 38.2 mm3, t=19.824, *p* < 0.001) and the iRFA+ATO group (326.3 ± 58.9 mm3, t=3.688, *p* = 0.006). Data are presented as mean ± SD. **(E)** Comparative analysis of tumor volumes on day 7 post-intervention among the four groups. Statistical significance was determined by one-way ANOVA followed by Bonferroni’s multiple comparisons test. **(F)** Kaplan-Meier survival curves depicting the survival outcomes of mice in the four experimental groups following intervention. Survival distributions differed significantly across groups (overall log-rank test, χ²=36.82, df=3, *p*<0.0001). Compared with iRFA alone, both iRFA+ATO and iRFA+LATO significantly reduced mortality hazard (HR = 0.050, 95% CI: 0.007–0.328, *p* = 0.0018 for both versus iRFA). Moreover, iRFA+LATO conferred a significantly lower hazard than iRFA+ATO (HR = 0.086, 95% CI: 0.015–0.502, *p* = 0.0064). **p < 0.01, ****p < 0.0001. (n = 5 per group).

**Figure 2:**
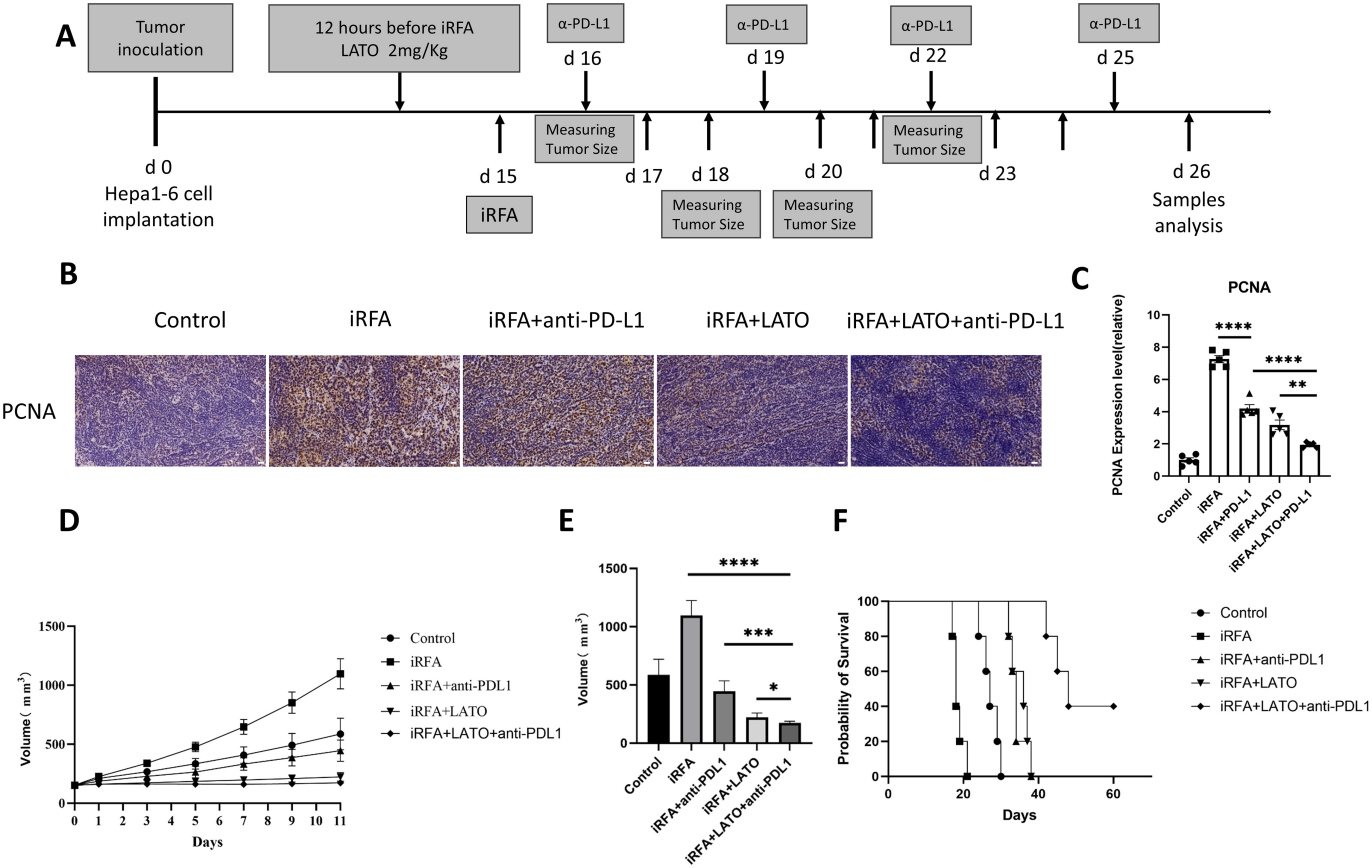
LATO synergistically potentiates the therapeutic efficacy of anti-PD-L1 antibody therapy following ablation, resulting in prolonged survival in tumor-bearing murine models. **(A)** Schematic illustration of the experimental protocol evaluating the combined effects of LATO and anti-PD-L1 antibody on iRFA-treated residual tumors. **(B)** Representative photomicrographs of PCNA immunohistochemical staining across five experimental groups (scale bar = 20 μm). **(C)** Quantitative analysis of PCNA-positive tumor cell ratios among the five treatment groups. Statistical significance was determined by one-way ANOVA followed by Bonferroni’s multiple comparisons test. **(D)** Longitudinal tumor volume monitoring in five groups post-iRFA. Mixed-design repeated-measures ANOVA revealed a significant main effect of Group [F(4,20)=75.232, *p* < 0.001, partial eta squared=0.938] and a significant Group x Time interaction [F(24,120)=76.126, *p* < 0.001, partial eta squared=0.938]. At day 11, the iRFA+LATO+anti-PD-L1 group (173.5 ± 13.0 mm3) showed significantly smaller tumor volume than iRFA alone (1096.5 ± 113.9 mm3, t=16.105, *p* < 0.001), iRFA+anti-PD-L1 (445.5 ± 81.0 mm3, t=6.635, *p* < 0.001), and iRFA+LATO (223.1 ± 32.6 mm3, t=2.825, *p* = 0.022). Data are presented as mean ± SD. **(E)** Comparative analysis of tumor volumes at day 7 post-treatment across the five groups. Statistical significance was determined by one-way ANOVA followed by Bonferroni’s multiple comparisons test. **(F)** Kaplan-Meier survival curves demonstrating the differential outcomes among the five treatment groups. Survival distributions differed significantly across groups (overall log-rank test, χ²=52.51, df=4, *p*<0.0001). Compared with iRFA alone, iRFA+anti-PD-L1, iRFA+LATO, and iRFA+LATO+anti-PD-L1 all significantly reduced mortality hazard (HR = 0.046, 95% CI: 0.007–0.318, *p* = 0.0018 for all versus iRFA). The combination of LATO with anti-PD-L1 further improved survival over anti-PD-L1 monotherapy (HR = 0.049, 95% CI: 0.007–0.333, *p* = 0.0021) and over LATO monotherapy (HR = 0.050, 95% CI: 0.007–0.328, *p* = 0.0018). *p < 0.05, **p < 0.01, ***p < 0.001, ****p < 0.0001. (n = 5 per group).

Data are expressed as mean ± SD or mean ± SEM. For all bar graphs and tumor growth curves, error bars represent mean ± SD. analyze Normality of distribution was assessed using the Shapiro–Wilk test; a *p* value > 0.05 was considered normally distributed. For comparisons among multiple groups with normally distributed data, one-way ANOVA was performed followed by Bonferroni-corrected *post-hoc* pairwise comparisons. For longitudinal data (tumor volumes in **Figures 1D**, **2D**; and serum biochemistry parameters in Section 2.7), mixed-design repeated-measures ANOVA was applied with Group as the between-subject factor and Time as the within-subject factor, to account for within-subject correlation across repeated measurements. Mauchly’s test of sphericity was performed; when violated, Greenhouse–Geisser corrected p values are reported. *Post-hoc* pairwise comparisons for repeated-measures data were conducted using independent samples t-tests with Bonferroni correction for multiple comparisons. For two-group comparisons with non-normal distribution, the Mann–Whitney U test was used. *p* < 0.05 was considered statistically significant. All statistical analyses were performed using GraphPad Prism 8. Survival curves were analyzed by the Kaplan–Meier method, and between-group differences were assessed by the log-rank test. Hazard ratios (HR) and 95% confidence intervals (CI) were calculated using the Mantel–Haenszel method with iRFA as the reference group. (*p < 0.05, **p < 0.01, ***p < 0.001, and ns for nonsignificant).

## 3 Results

### 3.1 LATO suppresses hypoxic HCC cell proliferation, invasion, migration, and oxidative stress adaptation

**Figure 3:**
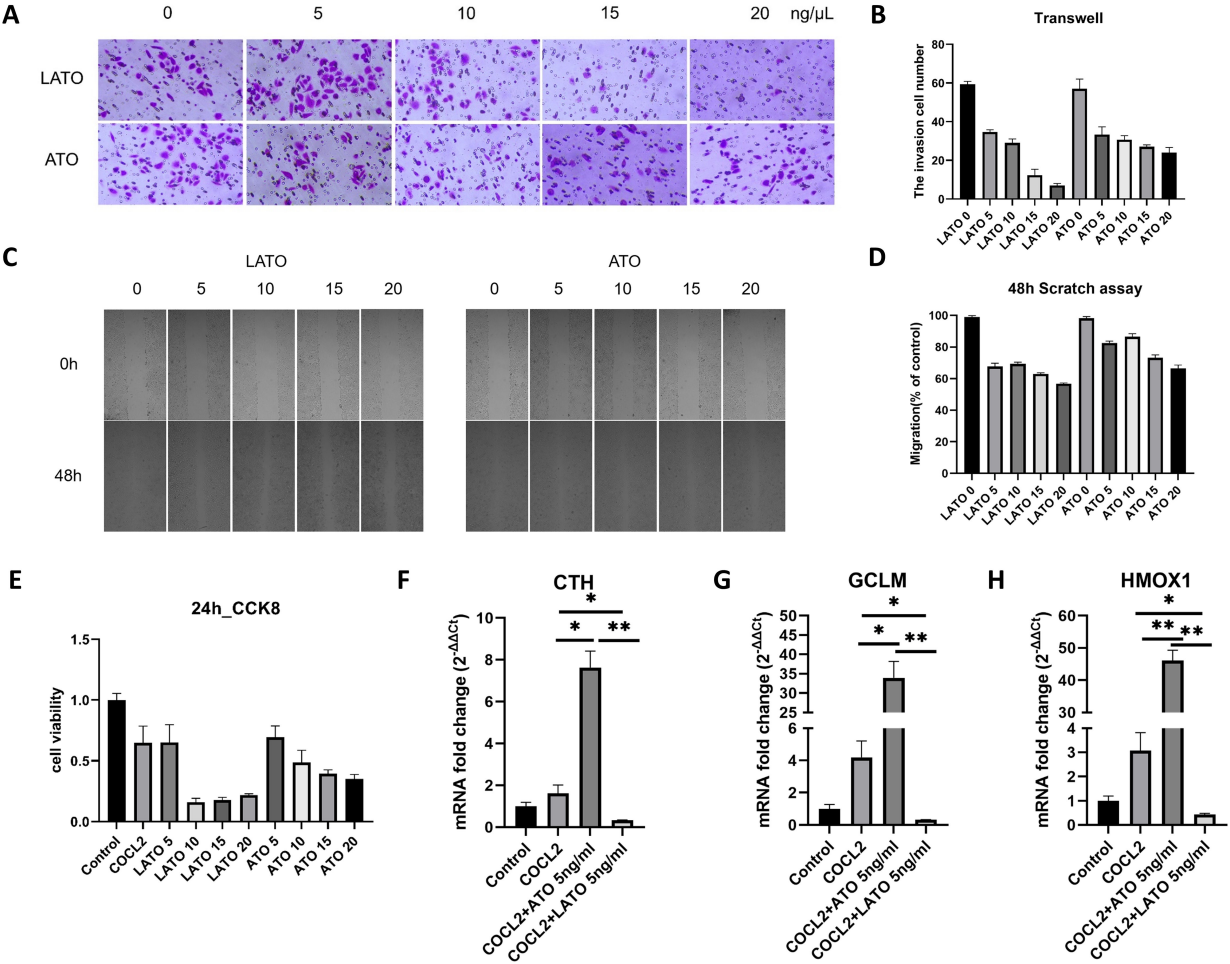
LATO inhibits hypoxic HCC cell proliferation, invasion, migration, and oxidative stress adaptation. **(A, B)** Evaluation of invasive capacity in Hepa1–6 cells. Statistical significance was determined by one-way ANOVA followed by Bonferroni’s multiple comparisons test. **(C)** Representative images of the scratch assay in Hepa1–6 cells at 0 h and 48 h. **(D)** Quantitative analysis of cell migration at 48 h; data are from three independent biological replicates (n = 3). Statistical significance was determined by two-way ANOVA followed by Šidák’s multiple comparisons test. LATO significantly reduced cell migration compared with ATO at 5, 10, 15, and 20 ng/ml (****p < 0.0001), whereas no significant difference was observed at 0 ng/ml (ns). **(E)** Measurement of proliferative activity in Hepa1–6 cells using CCK8 assay. Statistical significance was determined by two-way ANOVA followed by Šidák’s multiple comparisons test. **(F)** Quantitative analysis of CTH mRNA expression levels in Hepa1–6 cells. Statistical significance was determined by one-way ANOVA followed by Bonferroni’s multiple comparisons test. **(G)** Quantitative analysis of GCLM mRNA expression levels in Hepa1–6 cells. Statistical significance was determined by one-way ANOVA followed by Bonferroni’s multiple comparisons test. **(H)** Quantitative analysis of HMOX1 mRNA expression levels in Hepa1–6 cells. Statistical significance was determined by one-way ANOVA followed by Bonferroni’s multiple comparisons test.*p < 0.05, **p < 0.01.

Both ATO and LATO directly affected Hepa1–6 cell invasive ability; invasive cell number dropped with higher concentration, and LATO had a stronger effect (**Figures 3A, B**). At 48 h, the scratch assay showed that LATO significantly reduced Hepa1–6 cell migration compared with free ATO at concentrations of 5–20 ng/ml (**Figures 3C, D**). CCK8 showed 5 ng/ml ATO/LATO inhibited Hepa1–6 proliferation (COCL_2_-simulated hypoxia), LATO had stronger effect; higher LATO concentration further inhibited proliferation (**Figure 3E**). qPCR (5 ng/ml ATO/LATO, COCL_2_ hypoxia) showed: vs control, CoCl_2_ group Hepa1–6 cell CTH, GCLM, HMOX1 mRNA up-regulated; vs CoCl_2_ group, 5 ng/ml ATO up-regulated their mRNA, while 5 ng/ml LATO significantly inhibited their mRNA (**Figures 3F–H**). These show LATO inhibited Hepa1–6 cell oxidative stress resistance under CoCl_2_ hypoxia, thus suppressing proliferation.

### 3.2 The application of LATO solution in mouse subcutaneous tumor models of HCC is safe

**Figure 4:**
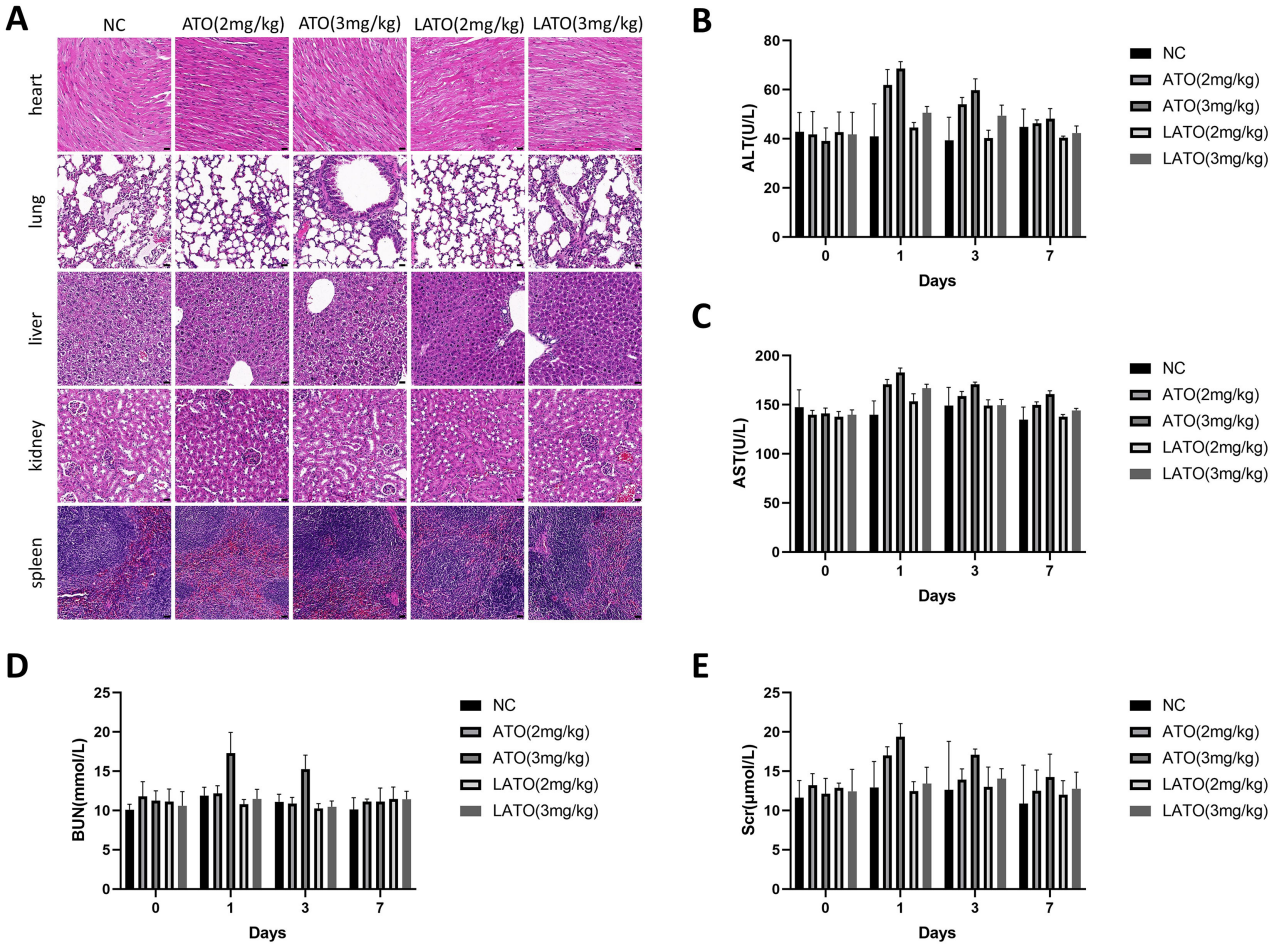
Safety evaluation of LATO solution in a murine subcutaneous hepatocellular carcinoma model. **(A)** Representative images of H&E-stained sections of heart, lung, liver, kidney, and spleen from tumor-bearing mice across groups at euthanasia (scale bar = 20 µm). **(B)** ALT, **(C)** AST, **(D)** BUN, and **(E)** Scr levels in mice at different time points. Data were analyzed by mixed-design repeated-measures ANOVA (Group × Time); *post-hoc* pairwise comparisons used Bonferroni correction.

After administering different concentrations of LATO and equivalent ATO in subcutaneous HCC mice, post-metabolic organ toxicity was assessed. All dose groups were well-tolerated; H&E staining showed no major organ abnormalities (**Figure 4A**). Mixed-design repeated-measures ANOVA (Group × Time) revealed significant Group × Time interactions for all four parameters (ALT, AST, BUN, Scr; all *p* < 0.05). *Post-hoc* analysis showed that 2 mg/kg LATO did not differ from NC at any timepoint for any parameter (all *p*>0.05), indicating minimal hepatorenal toxicity. By contrast, ATO 2 mg/kg and 3 mg/kg showed transient elevations in ALT, AST, BUN, and Scr at days 1 and/or 3 versus NC (all *p* < 0.05). Thus 2 mg/kg ATO/LATO was used for subsequent incomplete ablation models.

### 3.3 Application of LATO attenuated tumor proliferation after iRFA and prolonged survival in tumor-bearing mice

PCNA IHC and positive cell ratio quantification showed: vs control, iRFA group residual tumor cell proliferation increased significantly; ATO/LATO significantly attenuated post-iRFA residual tumor proliferation, LATO had stronger anti-proliferation effect (p<0.0001) (**Figures 1B, C**). Among 4 groups, iRFA group had fastest residual tumor growth and shortest survival; ATO/LATO reduced post-iRFA residual tumor growth rate and prolonged survival, LATO had strongest inhibitory effect (**Figures 1D–F**).

### 3.4 LATO can promote the infiltration of immune cells and inhibit tumor vascular proliferation

**Figure 5:**
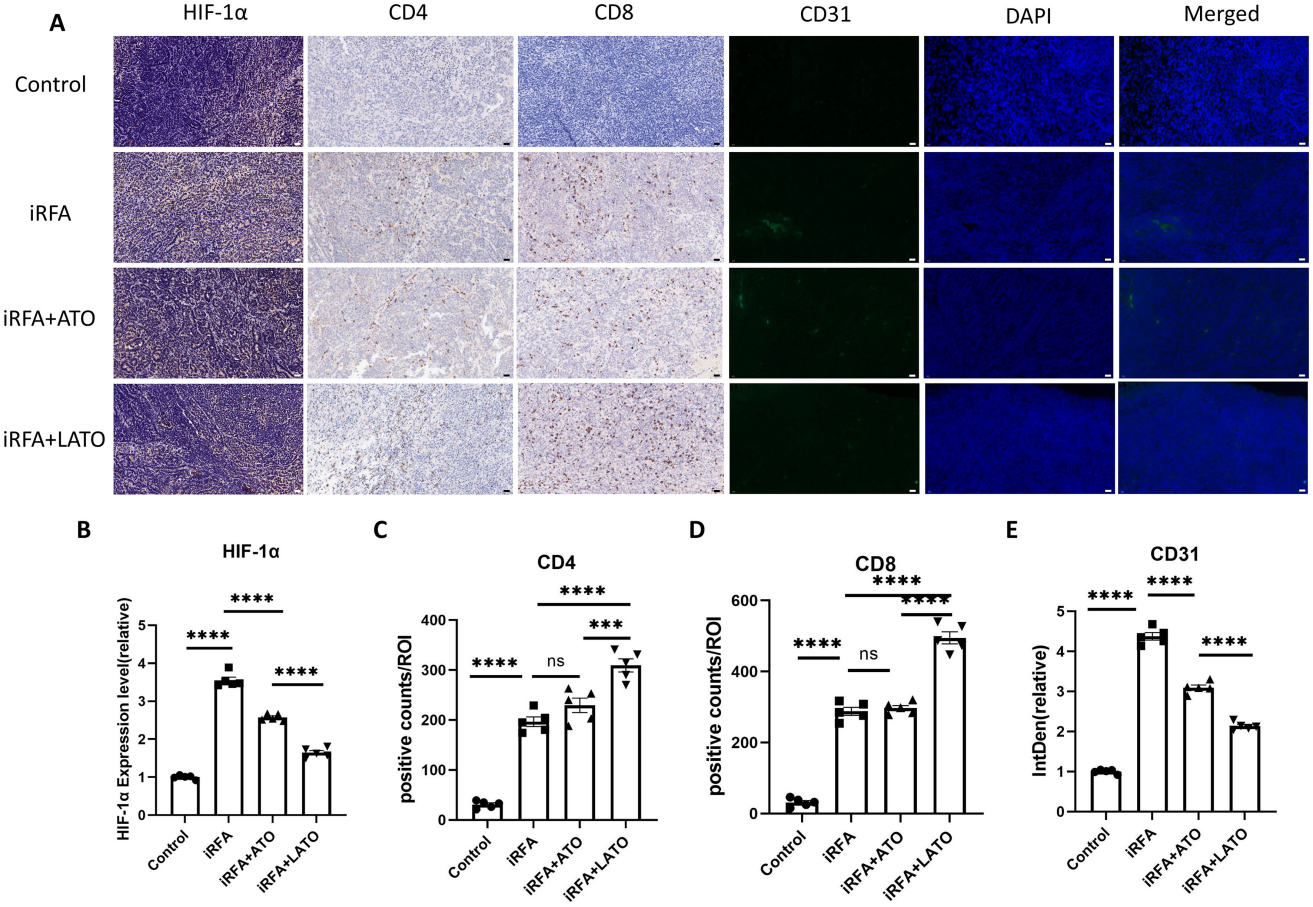
LATO enhances immune cell infiltration and suppresses tumor angiogenesis. **(A)** Representative immunohistochemical (IHC) staining images of HIF-1α, CD4^+^, CD8^+^, and CD31 expression across the four experimental groups (scale bar = 20 μm). **(B)** Quantitative analysis of cumulative optical density for HIF-1α expression in the four experimental groups. **(C)** Statistical quantification of CD4^+^ T-cell infiltration in the four experimental groups. **(D)** Statistical quantification of CD8^+^ T-cell infiltration in the four experimental groups. **(E)** Quantitative analysis of cumulative optical density for CD31 expression, a marker of vascular endothelial cells, in the four experimental groups. Statistical significance was determined by one-way ANOVA followed by Bonferroni’s multiple comparisons test. **p < 0.01, ***p < 0.001, ****p < 0.0001, ns=no significance. (n = 5 per group).

HIF-1α IHC and cumulative positive absorbance quantification showed: vs control, iRFA group residual tumor HIF-1α up-regulated significantly; ATO/LATO significantly down-regulated post-iRFA residual tumor HIF-1α, LATO group had greater decrease (**Figures 5A, B**). IHC (immune cell infiltration) showed: vs control, post-ablation residual tumor immune cell (CD4^+^, CD8^+^) infiltration increased (p<0.0001) (**Figures 5A, C, D**); LATO further enhanced residual tumor immune cell infiltration. CD31 IHC and cumulative fluorescence intensity quantification showed: vs control, iRFA group residual tumor angiogenesis increased (p<0.0001); ATO/LATO significantly attenuated post-iRFA residual tumor angiogenesis, LATO had stronger anti-angiogenesis effect (p<0.0001) (**Figures 5A, E**).

### 3.5 LATO synergistically enhanced anti-PD-L1 antibody therapy after ablation and prolonged survival in tumor-bearing mice

Relative PCNA expression was significantly lower in the iRFA + anti-PD-L1 group than in the iRFA group, indicating that anti-PD-L1 antibody treatment reduced the proliferative capacity of residual tumors after iRFA (**Figures 2B, C**). Compared with the iRFA + anti-PD-L1 group, the iRFA + LATO + anti-PD-L1 group showed a further decrease in PCNA expression, suggesting that LATO synergistically enhanced the anti-tumor effect of anti-PD-L1 therapy. Among the five groups, the iRFA group exhibited the most rapid tumor growth and the shortest survival. While either anti-PD-L1 or LATO alone slowed tumor growth and extended survival after iRFA, the combination of LATO with anti-PD-L1 resulted in the greatest suppression of tumor growth and the longest survival time (**Figures 2D–F**).

### 3.6 LATO combined with anti-PD-L1 antibody can promote the infiltration of immune cells in residual tumors and the expression of inflammatory factors in residual tumors

**Figure 6:**
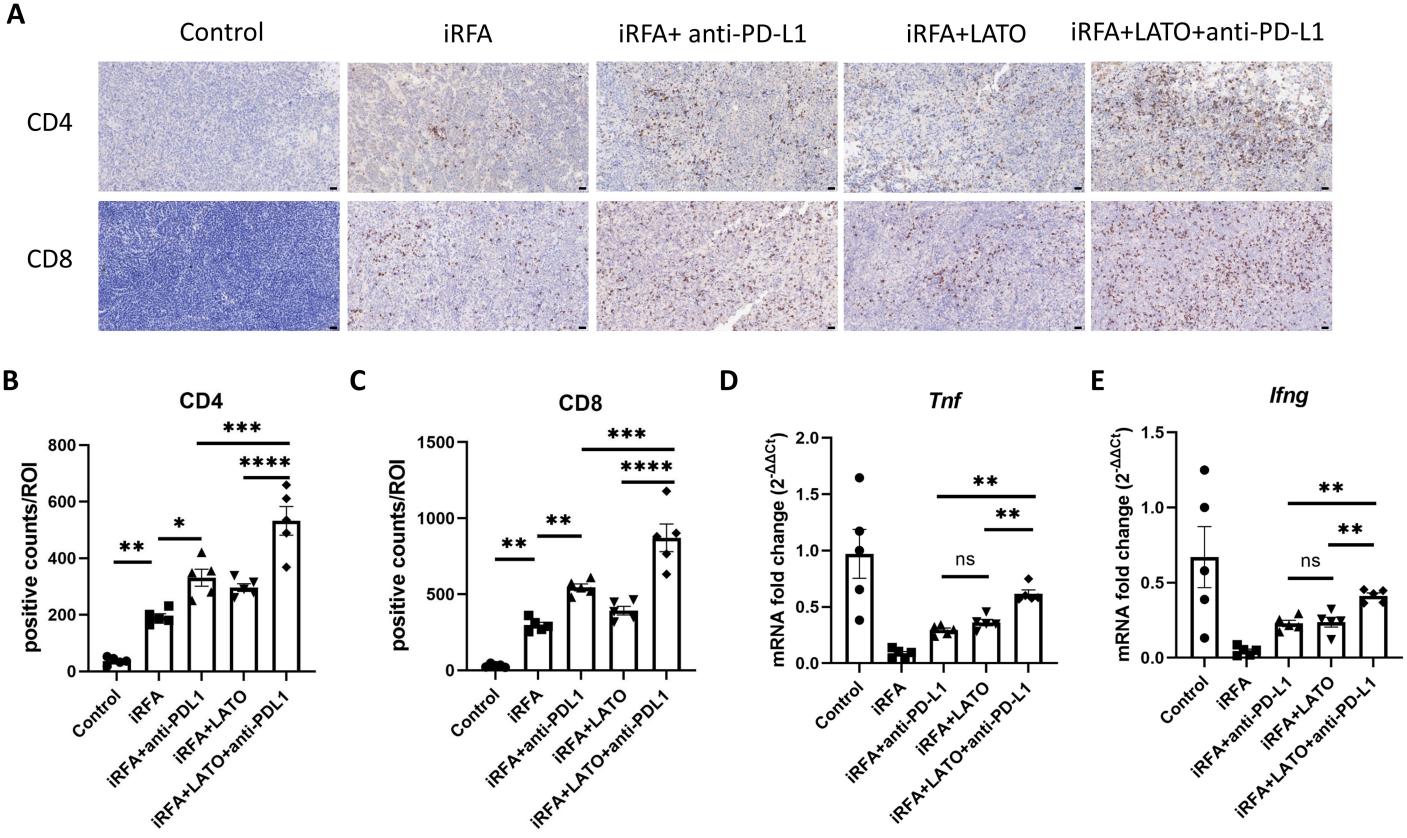
The combination of LATO with anti-PD-L1 antibody significantly enhances immune cell infiltration and upregulates inflammatory cytokine expression in residual tumors. **(A)** Representative immunohistochemical micrographs demonstrating CD4^+^ and CD8^+^ T cell infiltration patterns across five experimental groups (scale bar = 20 μm). **(B, C)** Quantitative analysis of CD4^+^ and CD8^+^ T lymphocyte infiltration densities in the five treatment groups. **(D)** Comparative mRNA expression profiles of tumor necrosis factor-alpha (Tnf) in residual tumors among experimental groups. **(E)** Differential interferon-gamma (Ifng) mRNA expression levels in residual tumors across treatment groups. Statistical significance was determined by one-way ANOVA followed by Bonferroni’s multiple comparisons test. *p < 0.05, **p < 0.01, ***p < 0.001, ****p < 0.0001. (n = 5 per group).

The infiltration of CD4^+^ and CD8^+^ T cells into residual tumors was evaluated by IHC (**Figures 6A–C**). Anti-PD-L1 treatment markedly increased the infiltration of both CD4^+^ and CD8^+^ T cells in post-ablation tumors (p< 0.001), an effect that was further enhanced by co-administration of LATO. Inflammatory cytokines such as TNF-α and IFN-γ, which can exert tumoricidal effects within the tumor microenvironment (TME) (20, 21), were also measured (**Figures 6D, E**). Compared with either iRFA + anti-PD-L1 or iRFA + LATO alone, the iRFA + LATO + anti-PD-L1 group exhibited significantly upregulated mRNA expression of Tnf and Ifng (p< 0.001 for both). Meanwhile, no significant difference in Tnf or Ifng expression was observed between the iRFA + anti-PD-L1 and iRFA + LATO groups. These results indicate that the combination of LATO and anti-PD-L1 promotes the expression of inflammatory cytokines such as TNF-α and IFN-γ in residual tumors, contributing to tumor eradication.

## 4 Discussion

Our findings demonstrated that LATO effectively suppressed the proliferation, invasion, migration, and oxidative stress resistance of HCC cells under hypoxic conditions, increase the ablation efficacy, and demonstrated that liposomes could increase the phagocytosis of liposomal drugs by tumor cells, with good safety in animal experiments. HIF-1α expression increased in residual tumor tissue after iRFA, which promoted tumor cell proliferation. Preoperative application of LATO combined with postoperative PD-L1 in RFA elicited maximal therapeutic benefit in inhibiting cell proliferation, promoting immunosuppressive cell infiltration, and promoting the expression of inflammatory factors in the residual tumor. Finally, it was confirmed that combined therapy could improve the efficacy of thermal ablation and improve the immune microenvironment. Mechanistically, the liposomal encapsulation enhances tumor-specific ATO delivery via the Enhanced Permeability and Retention (EPR) effect, increasing intratumoral drug bioavailability and prolonging its pharmacodynamic action. Within the hypoxic TME, ATO suppresses HIF-1α transcriptional activity or promotes its protein degradation, thereby disrupting the downstream expression of pro-angiogenic and immune-suppressive effectors.

As the tumor diameter increases, the rate of complete necrosis after RFA gradually decreases (22). iRFA can promote the rapid growth of residual cancer through autophagy and epithelial-mesenchymal transition, and can also promote HCC metastasis (23). Kong et al. (6) showed that incomplete ablation of liver tumors can promote HIF-1α expression in residual tumor tissues, lead to the opening of HIF-1α/vascular endothelial growth factor (VEGF) pathway, overexpress VEGF, promote angiogenesis in residual tumor tissues, and accelerate the progression of residual tumor tissues. LATO counteracts this hypoxic adaptation by enriching ATO in residual tumor tissues and selectively inhibiting the HIF-1α/VEGF transcriptional program, thereby preventing the angiogenic switch that drives post-ablation tumor progression. In this study, the increased expression of PCNA and HIF-1α 11 days after RFA means that the changes in the tumor microenvironment brought about by incomplete ablation will significantly enhance the growth and invasion of residual tumors, which is consistent with the findings that the hypoxic microenvironment after RFA will promote the growth of residual tumors obtained by Nijkamp et al (24, 25).

In this study, preoperative LATO administration significantly downregulated PCNA, HIF-1α, and CD31 in residual tumors post-iRFA, indicating that LATO remodels the tumor microenvironment through a HIF-1α–centered mechanistic axis. We propose a dual mechanism whereby LATO-mediated HIF-1α suppression coordinately attenuates angiogenic drive and relieves adaptive immune resistance. First, HIF-1α inhibition reduces VEGF-dependent aberrant neovascularization, leading to a decrease in immature, hyperpermeable CD31^+^ vessels. Rather than compromising perfusion, this vascular pruning favors a “normalization” phenotype characterized by reduced vascular permeability, improved pericyte coverage, and enhanced intratumoral blood flow, which collectively alleviates hypoxia and facilitates CD4^+^ and CD8^+^ T-cell extravasation into the tumor bed. This reconciles the apparent paradox of decreased CD31^+^ vessel density yet increased immune cell infiltration observed in the LATO group. Second, mechanistically, HIF-1α functions as a direct transcriptional activator of PD-L1; its suppression by LATO downregulates PD-L1 expression at the transcriptional level, thereby dismantling a critical hypoxia-adaptive immune checkpoint and lowering the threshold for T-cell–mediated antitumor immunity. This HIF-1α/PD-L1 axis provides the molecular link between LATO’s metabolic intervention and the enhanced immune infiltration phenotype. Functionally, both ATO and LATO attenuated residual tumor growth and prolonged survival relative to iRFA alone, with LATO exerting the strongest effect—consistent with the notion that ATO inhibits post-ablation recurrence by normalizing the tumor microenvironment and suppressing pathological angiogenesis, as previously reported by Lew et al (26). The superior efficacy of LATO over free ATO likely reflects enhanced tumor deposition and prolonged intratumoral retention via the EPR effect, a pharmacokinetic advantage that we and others have documented with liposomal formulations (27). The superior efficacy of LATO over free ATO can be systematically explained by the advantages of liposomal encapsulation. Liposomes passively target tumors via the EPR effect, achieving higher intratumoral drug concentrations than non-specifically distributed free ATO (28, 29). This encapsulation also profoundly improves pharmacokinetics by shielding ATO from rapid systemic clearance and protein binding, thereby prolonging circulation time, increasing the AUC, and critically, reducing the dose-limiting cardiotoxicity characteristic of ATO—as directly reflected by the improved safety and efficacy of LATO over equivalent-dose ATO in our tolerability and therapeutic experiments (29). Consequently, the enhanced and sustained tumor deposition enabled by the liposomal carrier allows more effective suppression of HIF-1α, which in turn disrupts downstream angiogenic and immunosuppressive pathways, ultimately amplifying the therapeutic benefit (30). Collectively, these findings establish LATO as a neoadjuvant TME-modulating agent that simultaneously targets the HIF-1α/VEGF angiogenic loop and the HIF-1α/PD-L1 immune-evasion axis to constrain residual tumor progression after iRFA.

Combining thermal ablation with immune checkpoint inhibitors (ICIs) represents an emerging strategy to improve HCC treatment efficacy (23). RFA not only achieves local tumor destruction but can also elicit systemic antitumor immunity through the “distant effect” (31), wherein *in situ* necrosis releases tumor-associated antigens (TAAs) that prime adaptive T-cell responses (32). Indeed, Liang et al. (33) demonstrated that RFA combined with PD-L1 blockade augments CD8^+^ T-cell cytotoxicity and reduces Treg infiltration in murine models. However, clinical response rates to anti–PD-1/PD-L1 monotherapy in HCC remain suboptimal (<25%) (34), partly because a substantial proportion of HCCs are immunologically “cold” and fail to mount effective intratumoral T-cell responses. We posit that this resistance is mechanistically rooted in the HIF-1α/PD-L1 axis activated by iRFA-induced hypoxia. Barsoum et al. (35) demonstrated hypoxia-driven PD-L1 upregulation in tumor cells, and Dai et al. (36) identified HIF-1α pathway activation as a key driver of PD-L1 expression in HCC. In our model, residual tumor hypoxia following iRFA stabilizes HIF-1α protein, which directly binds the PD-L1 promoter to transcriptionally upregulate PD-L1, thereby inducing T-cell exhaustion and adaptive immune resistance. Neoadjuvant LATO disrupts this axis by suppressing HIF-1α, lowering the baseline transcription of PD-L1 and reducing the immunosuppressive threshold. Consequently, when anti–PD-L1 antibody is administered post-RFA, it encounters a less immunosuppressive microenvironment and more efficiently blocks residual PD-1/PD-L1 interactions, restoring CD4^+^ and CD8^+^ T-cell cytotoxic function. The marked upregulation of TNF-α and IFN-γ mRNA in the iRFA + LATO + anti–PD-L1 group confirms the re-establishment of a Th1-dominant antitumor immune response. This sequential mechanism—RFA-induced antigen release followed by LATO-mediated HIF-1α/PD-L1 axis suppression and subsequent PD-L1 checkpoint blockade—provides a coherent molecular rationale for the synergistic efficacy observed. Clinically, trials combining RFA or MWA with nivolumab or pembrolizumab have already improved responder rates from ~10% to 24% (37), underscoring the translational promise of integrating locoregional ablation with ICIs. Our data extend this concept by demonstrating that preoperative LATO priming can further sensitize residual tumors to anti–PD-L1 therapy, offering a mechanistic foundation for optimized perioperative combination regimens in HCC (38).

In clinical practice, iRFA is often followed by adjuvant therapies. Our findings suggest that preoperative LATO may serve as a proactive strategy to sensitize residual tumors to subsequent immune checkpoint inhibition, potentially improving outcomes over conventional approaches. To further investigate this, anti-PD-L1 antibody therapy was included in the study, with LATO combined with anti-PD-L1 after RFA. Compared with the iRFA group, PCNA expression was significantly lower in the iRFA + anti-PD-L1 group, indicating that anti-PD-L1 attenuated residual tumor proliferation. Moreover, the iRFA + LATO + anti-PD-L1 group showed a further reduction in PCNA expression compared to iRFA + anti-PD-L1 alone, suggesting that LATO synergistically enhances the anti-tumor effect of anti-PD-L1 after iRFA. Although no significant difference in *Tnf* or *Ifng* mRNA was observed between the iRFA + anti-PD-L1 and iRFA + LATO groups, we speculate that LATO in the latter group may downregulate PD-L1 by inhibiting the HIF-1α pathway post-RFA, thereby promoting CD4^+^ and CD8^+^ T-cell infiltration and enhancing tumor killing. Thus, the combination of LATO and anti-PD-L1 may boost the efficacy of anti-PD-L1 after RFA, increase inflammatory factor secretion, and ultimately inhibit tumor growth while prolonging survival. Biologically, these findings establish neoadjuvant LATO administration as a TME-targeting strategy that converts an immunologically ‘cold’ or ‘excluded’ residual tumor into an inflamed (‘hot’) phenotype by disrupting the HIF-1α/PD-L1 axis. This metabolic-immune priming may expand the responder population for ICI therapy and provide a translational rationale for perioperative combination regimens in HCC.”

Additionally, LATO-mediated suppression of CTH, GCLM, and HMOX1 expression suggests that LATO may impair the oxidative stress adaptation of residual HCC cells under hypoxia. This metabolic vulnerability, coupled with immune microenvironment remodeling, may synergistically constrain residual tumor progression.

This study has several limitations. First, the subcutaneous tumor model employed may not fully recapitulate the hepatic immune microenvironment or clinical progression of hepatocellular carcinoma. Second, PD-L1 expression following iRFA was not directly measured, thus the proposed mechanism requires further validation. Third, while the study focused on T-cell infiltration and hypoxia markers, a more comprehensive analysis of myeloid cells—such as macrophages and MDSCs—as well as dendritic cells is needed to fully characterize the tumor immune microenvironment. Additionally, the relatively small sample size may limit the statistical power of the survival analyses, and direct evidence supporting the role of the HIF-1α/PD-L1 axis in LATO’s mechanism remains lacking. Future studies employing orthotopic or spontaneous HCC models, with larger cohort sizes, expanded detection metrics, and optimized dosing regimens of LATO, are warranted to validate these findings and further evaluate the impact of this combination strategy on distant metastasis and immune modulation.

## 5 Conclusion

This study represents the initial application of LATO in animal iRFA models. The findings suggest that LATO may help inhibit the invasiveness of residual tumors post-iRFA, potentially improve the efficacy of HCC ablation, and enhance PD-L1-mediated antitumor immune responses by modulating the post-ablation immune microenvironment. These observations could offer preliminary insights and future directions for combining local therapy with immunotherapy in HCC treatment.

## Data Availability

The raw data supporting the conclusions of this article will be made available by the authors, without undue reservation.
