# Supplementary material for: Mechanism of liposome arsenic trioxide regulating tumor microenvironment and sensitizing tumor immune response after radiofrequency ablation
Source: Front Immunol. 2026 Jun 29;17:1776060. doi: 10.3389/fimmu.2026.1776060 (PMC13357814; doi:10.3389/fimmu.2026.1776060)
Supplement: Supplementary file 1 [file DataSheet1.docx]

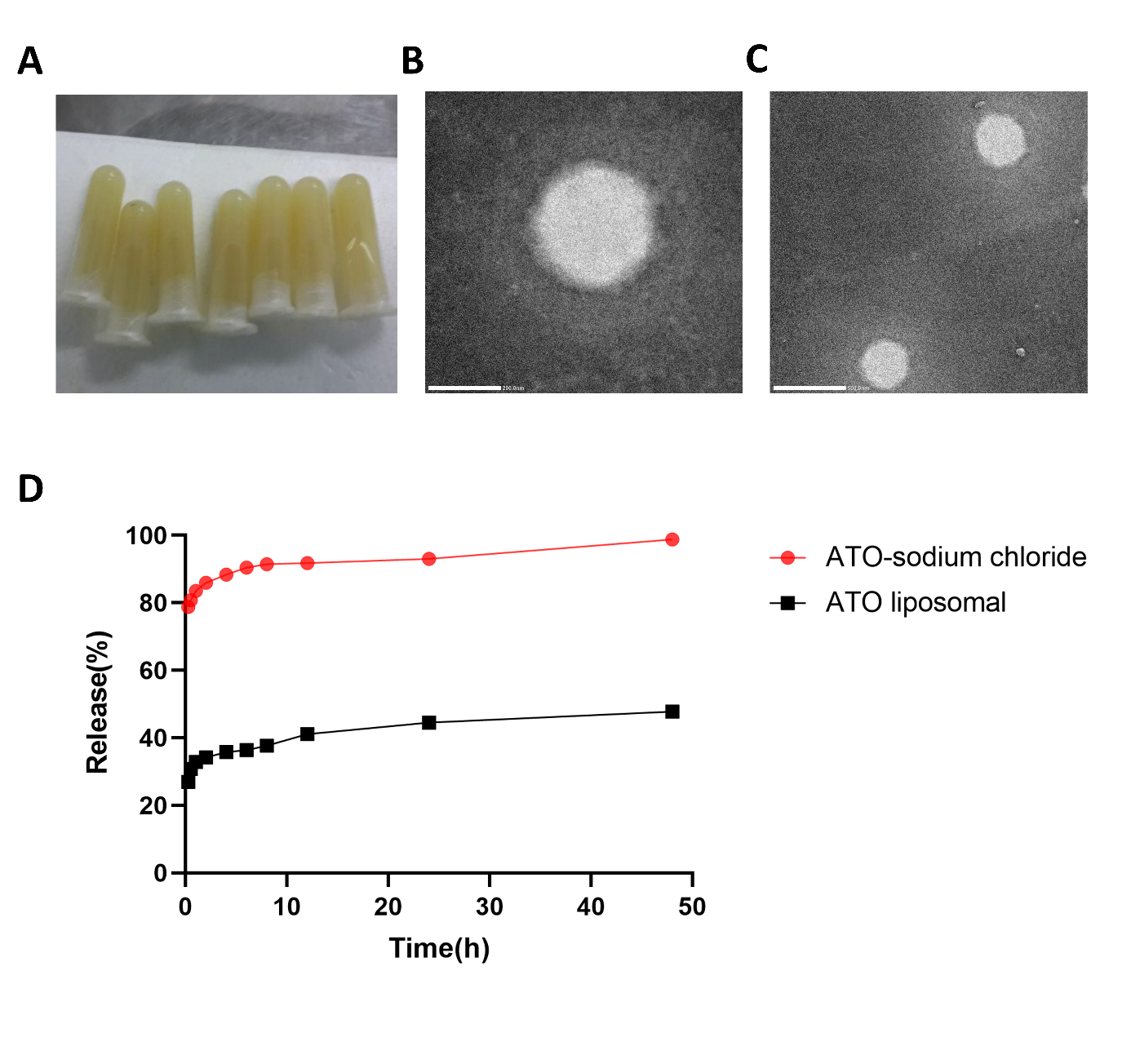
The prepared LATO solution appeared as a milky white liquid with no precipitate at the bottom (Fig. S1A). Transmission electron microscopy revealed that the liposomes exhibited smooth and round surfaces and were uniformly distributed in the medium (Fig. S1B, Fig. S1C). The release of the ATO bulk drug was relatively rapid, being essentially complete within 6 h. LATO showed an initial fast release, reaching 35.2% at 2 h, followed by a relatively stable phase from 2 to 8 h. A slightly higher release of 38% was observed between 8 and 24 h, with a slower release in the later stage. Compared with the ATO solution, LATO demonstrated a significant sustained-release effect. The cumulative release at 48 h was only about 44%, with no obvious burst release (Fig. S1D). The liposomes exhibited a uniform particle size distribution, with an average particle size of approximately 173 nm. The mean encapsulation efficiency of LATO was determined to be 80%. The Zeta potential of LATO was measured as 10.6 mV. The liposomes showed a homogeneous particle size distribution, an average particle size of around 173 nm, and a polydispersity index (PDI) of 0.175.

**Figure S1** Characterization of LATO. (A) Photograph of LATO appearance; (B, C) Transmission electron microscopy images of LATO; (D) Drug release profiles of LATO and ATO solution over time.
